# Driving a Semiautonomous Mobile Robotic Car Controlled by an SSVEP-Based BCI

**DOI:** 10.1155/2016/4909685

**Published:** 2016-07-26

**Authors:** Piotr Stawicki, Felix Gembler, Ivan Volosyak

**Affiliations:** Faculty of Technology and Bionics, Rhine-Waal University of Applied Sciences, 47533 Kleve, Germany

## Abstract

Brain-computer interfaces represent a range of acknowledged technologies that translate brain activity into computer commands. The aim of our research is to develop and evaluate a BCI control application for certain assistive technologies that can be used for remote telepresence or remote driving. The communication channel to the target device is based on the steady-state visual evoked potentials. In order to test the control application, a mobile robotic car (MRC) was introduced and a four-class BCI graphical user interface (with live video feedback and stimulation boxes on the same screen) for piloting the MRC was designed. For the purpose of evaluating a potential real-life scenario for such assistive technology, we present a study where 61 subjects steered the MRC through a predetermined route. All 61 subjects were able to control the MRC and finish the experiment (mean time 207.08 s, SD 50.25) with a mean (SD) accuracy and ITR of 93.03% (5.73) and 14.07 bits/min (4.44), respectively. The results show that our proposed SSVEP-based BCI control application is suitable for mobile robots with a shared-control approach. We also did not observe any negative influence of the simultaneous live video feedback and SSVEP stimulation on the performance of the BCI system.

## 1. Introduction

The idea of remotely operated vehicles without the necessity of manual supervision concerns humankind since the end of 19th century. The first public demonstration of a wireless submarine was performed by Nicola Tesla in 1898 [1]. The first automatically operated vehicles utilizing autonomous behavior were* Elmer* and* Elsie*, built by neurobiologist William Grey Walter in Bristol, England, in the late 1940s [2]. Since then, numerous areas of application for robot vehicles (military, environment, and healthcare) have emerged. In healthcare applications, robotic vehicles are primarily developed as assistive devices for severely disabled or paralyzed patients or patients suffering from a stroke [3]. For some people with severe motor impairments like amyotrophic lateral sclerosis (ALS) or spinal cord injury (SCI), traditional control interfaces, such as a 2-axis joystick, a keyboard, or a mouse, are not suitable, as they require precise movement control over the limbs, which in their case is not possible due to nerve cells degradation or spinal nerve damage. For these people, accurate control interfaces can be realized through modern technologies such as brain-computer interfaces (BCIs). The term brain-computer interface (BCI) or brain-machine interface (BMI) represents a range of technologies developed intensively over the past 20 years. BCIs introduce additional communication pathways that translate brain signals directly into computer commands (without the use of muscles or nerves) and therefore provide people with minor to severe disabilities with a new opportunity to enhance their quality of life; for some people, BCIs offer a new way of communicating and, for others, a new way to control their environment (e.g., wheelchair, prosthesis) [4–6].

Service robots designed to assist paralyzed people are a popular research topic in the literature on BCIs [7]. Technical solutions for such robots are developed with different BCI paradigms. In the last decade, numerous robot control BCI applications were developed.

Ron-Angevin et al. used a SMR-based two-class BCI application to choose between four direction commands in order to control a robot [8]. Three out of four subjects were able to control their BCI robot. Tonin et al. developed a shared control of two mental states' brain-machine interface for steering a telepresence robot located at a distance of 100 km [9]. All four subjects (two healthy and two with motor disabilities, myopathy) successfully controlled the robot with a similar performance. Huang et al. used an event-related desynchronization/synchronization of electroencephalogram signals to control a 2D, constantly moving, virtual wheelchair [10]. Four out of five healthy subjects reached mean target hit rates of 87.5% and 100% with motor imaginary in two virtual games.

Several studies used the P300 paradigm (another commonly used BCI approach using the 300 ms component of an evoked potential). Chella et al. presented a Museum Robotic Guide GUI for P300-based BCI developed at the San Camillo Institute [11]. The user can choose between two robots (Pioneer3 or PeopleBot), each located in a different place, to visit the museum. Escolano et al. reported a P300-based telepresence system that enables the user to be present in remote environments through a mobile robot using a shared-control strategy [12].

Reliable BCIs can also be realized with steady-state visual evoked potentials (SSVEPs). SSVEPs are the continuous brain responses elicited at the visual and parietal cortical areas under visual stimulation with a specific constant frequency [13]. Shortly, when looking at a light source that flickers with a constant frequency, the corresponding brain signals are measured with the EEG from the occipital cortex. As the specific stimulation frequencies are known a priori, the stimuli the user focuses its gaze on can be detected through classification algorithms that detect the stimulation frequency with the strongest amplitude, that is, through analyzing the signal to noise ratios.

Visual evoked potentials (VEP) recorded in the 1960s by Regan [14] and early tested in the 1980s and 1990s extended our knowledge about the human brain. For example, as early as 1979, Frank and Torres evaluated the use of visual evoked potentials in diagnosis of “cortical blindness” in children [15]. VEP can also support the diagnosis of multiple sclerosis [16].

At the beginning of the 21st century, the progress of computing power and technology allowed the extensive research and development of SSVEP and its use in BCI. A recent review of visual and auditory BCIs can be found in [17]. Various applications like spelling interfaces [18] and control applications for a prosthesis [19] or for navigation [20] can be implemented with the SSVEP paradigm. In a large demographic study with 86 subjects, Volosyak et al. presented a four-class, SSVEP-based BCI with LED stimulation [21]. 84 subjects reached a mean (SD) accuracy and ITR of 92.26 (7.82)% and 17.24 (6.99) bits/min, respectively, with stimulation at 13, 14, 15, and 16 Hz (medium frequency set). Only 56 subjects were able to operate the BCI at high frequencies (34, 36, 38, and 40 Hz), reaching a mean accuracy and ITR of 89.16 (9.29)% and 12.10 (7.31) bits/min. Zhao et al. introduced a three-layer decision tree for humanoid robot BCI interaction [22]. The SSVEP stimulation was presented on a 17′′ LCD monitor. Four boxes were flickering at 5, 6, 8, and 10 Hz with an additional 17 Hz red LED on top of the monitor. Five young subjects tested the developed software platform. The mean performance accuracy achieved by four of them was approximately 88%. Guneysu and Levent Akin presented a four-class SSVEP-based BCI with LED stimulation at 7, 9, 11, and 15 Hz; an Emotiv EPOC headset was used for EEG data acquisition and MATLAB software (FFT with Gaussian model) was used for dominant frequency extraction [23]. Three subjects achieved an average performance of approximately 68% at a distance of 22 cm from the LEDs. Holewa and Nawrocka presented an SSVEP-based BCI for Lego Mindstorm robot control with an Emotiv EPOC headset [24]. For visual simulation, four LED-based panels with stimulation frequencies of 28, 30, 32, and 34 Hz were used. The panels were additionally covered with different color filters. Three subjects operated this setup with a mean accuracy and ITR of 73.75% and 11.36 bits/min. Kwak et al. presented an SSVEP-control system for the exoskeleton REX [25]. Five LEDs blinking at 9, 11, 13, 15, and 17 Hz were used to select between the exoskeleton commands forward, left, standing, right, and sitting, respectively. Three subjects achieved a mean accuracy of 99.16%. In an f-VEP based BCI, each target is flashing at a different constant frequency, causing a periodic sequence of evoked responses with the same fundamental frequency as that of the flickered stimulus as well as its harmonics [26], and in a c-VEP BCI usually a pseudorandom m-sequence phase-shifted between targets is flashing (m-sequence with an autocorrelation function which is a very close approximation to a unit impulse function, and it is nearly orthogonal to its time lag sequence) [26]. Kapeller et al. evaluated a frequency-coded f-VEP (8.57, 10, 12, and 15 Hz) system and code modulated c-VEP (63-bit pseudorandom m-sequence) as a continuous robot steering input with video feedback of the robot movement [27]. Eleven healthy subjects achieved a maximum accuracy of 91.36% and 98.18% for f-VEP and c-VEP, respectively, in an online accuracy test run without zero class. Wang et al. presented a handmade remote control electrical car, which was driven using an SSVEP stimulation with blue LEDs (controlled via ATmega8L microcontroller) [28]. Combining Average and Fast Fourier Transform calculations, they achieved an average accuracy of 85, 91, 94, 98, and 100% for the different trial lengths of 0.5, 1, 2, 4, and 8 seconds, respectively. The EEG signal was recorded from electrodes *O*_1_ and *O*_2_ of the international 10–20 system. Gergondet et al. used SSVEP stimulation with approximated frequencies (frequency coding, method originally proposed by Wang et al. [29]) for steering a humanoid robot (HRP-2) [30]. Four stimulation arrows (6, 8, 9, and 10 Hz) were presented on a 17′′ LCD screen with 60 Hz vertical refresh rate. Also presented in a background area of the screen was a live video stream from the robot's point of view. Their research focused on the impact of the background, dynamic compared to static. Diez et al. tested an SSVEP-driven Pioneer 3-DX mobile robot equipped with a video camera for live feedback and an obstacle avoidance ultrasonic sensor system [31]. The high frequency (37, 38, 39, and 40 Hz) visual stimulation was generated with an FPGA card and stimulation modules (LED-based) were placed on the outer edge of the monitor in a top, left, right, and bottom arrangement, and the camera image was displayed live on the monitor. BCI commands were classified using a sliding window FFT method. Seven participants navigated the robot to two different office locations. Some recent studies combine at least two of the BCI paradigms to realize a so-called hybrid BCI. Choi and Jo combined the SSVEP with event-related desynchronization (ERD, typical SMR-based BCI) for the navigation/exploration of a humanoid robot, with additional P300 BCI for interaction (object recognition, decision) [32]. They also tested and evaluated the use of a low-cost BCI system. The ERD and SSVEP BCIs were two-class systems; the number of classes for the P300 BCI varied (2 to 4, depending on the detected objects). Five subjects used a low-cost Emotiv EPOC headset to perform the experiment. The overall accuracies achieved by the ERD and SSVEP protocols were 84.6% and 84.4%, respectively; the P300 protocol reached an accuracy of 91% for two objects and 89.5% for four objects through the robot's camera. The scored ITR value for the navigation/exploration task was 18.1 bits/min and for the objects recognition task 21 bits/min. Authors reported that switching between SSVEP and ERD caused a drop in accuracy down to 73%. The robot's point of view was shown in the middle of the monitor (live feedback), the SSVEP stimulation was shown at the sides, and the P300 was shown directly in the live feedback of the robot's view. Tidoni et al. presented a study where nine subjects (located in Italy) remotely controlled a humanoid robot (HRP-2, located in Japan) [33]. Subjects tested a synchronous, asynchronous audio-visual feedback and evaluated whether a mirror reflection of the robot (in its field of view) improved the performance of an SSVEP-based BCI with video feedback. The main task was to navigate the robot and to move a bottle from one table to another. A six-class SSVEP-based BCI was used, the stimulation and the video feedback were presented on the same screen. The flashing frequencies were 6, 8, 9, 10, and 14 Hz, with an additional zero class when the subject did not gaze at any target. Due to the remote distance, the feedback action was visible to the subject after approximately 800 ms. Zhao et al. compared a four-class SSVEP-based BCI to a six-class P300-based BCI by evaluating achieved control accuracies of robot navigation tasks [34]. For both systems (SSVEP and P300), the modular platform Cerebot was used and stimuli were presented on a LCD monitor. Seven subjects took part in the experiment and achieved a mean accuracy and ITR of 91.3% and 18.8 bits/min for the SSVEP approach; for the P300 approach, 90.3% and 24.7 bits/min were achieved.

Rutkowski et al. implemented a robot control application using tactile BCIs [35]. They tested two tactile stimulation configurations: a pin-pressure BCI (arranged in a 3 × 3 matrix) and a full body vibrotactile BCI (vibrotactile generators placed on subjects backs, shoulders, arms, and legs). A humanoid NAO robot was navigated using six predefined commands. Five subjects could control the robot using the pin-pressure BCI and three subjects successfully tested the full body vibrotactile BCI. The authors demonstrate the reliability of the tactile BCI paradigm for robot navigation.

All listed studies have to deal with the main engineering problems regarding BCI technologies. EEG-based BCIs are relatively slow and can sometimes be unreliable. Maximizing the speed of a BCI while still maintaining high accuracy leads to certain limitations in the number of targets and in system speed (target selection). Moreover, it is often reported that a certain number of subjects are not able to control BCIs (so-called BCI illiterates, e.g., [21, 36]). We chose the SSVEP paradigm, as it is reported to be among the fastest and most reliable BCI strategies, with short or no training time needed [13]. In our recent study, after five years of research, we developed an SSVEP calibration software (SSVEP Wizard) for LCD monitors that determines SSVEP key parameters for each subject [37]. The selected stimulation frequencies are those with the highest SSVEP response in the visual cortex. We have shown that, with optimized parameters, the SSVEP BCI literacy rate can reach 100%. This calibration software is also used here to determine and optimize the user-specific SSVEP parameters. Some SSVEP-based applications described problems regarding video feedback. The research of Gergondet et al. was focused on the impact of the background (dynamic compared to static) on the BCI performance. They reported that a dynamic background negatively affected the SSVEP stimulation [30]. Kapeller et al. noted a slight decrease in performance for an SSVEP-based BCI with background video and small targets [27]. In the approach presented by Diez et al., the stimulation was separated from the video feedback stream (outer LEDs), but no negative influence of the live feedback was reported [31].

SSVEP-based BCI systems for robot control usually use four or five control commands. A higher degree-of-freedom control is difficult to realize, because multitarget BCIs usually have a lower accuracy. A countermeasure to the limitations in degrees of freedom can be a shared-control approach; that is, the robot can share some specific control commands with the human user which allows for the faster execution of complex tasks. The so-called shared-control principle was initially explored for BCI-controlled wheelchairs and recently applied to BCI telepresence [12]. BCIs with only a few targets are more robust and therefore more suitable for control applications. There are already numerous existing BCI technologies that patients use at their homes [38, 39]. However, for the last decade, publications about SSVEP-based BCIs have been sparse. The PubMed database shows 4200 results for the search term “Brain-computer interface”/“Brain-machine interface” but only 185 results for the term “SSVEP”. With this paper, we would like to contribute to the research of SSVEP-based BCIs.

In this paper, we present a four-class, SSVEP-based, BCI control application with live video feedback. We also present a self-made, low-cost, semiautonomous mobile robotic car (MRC) with live video feedback, controlled by a BCI application. Unlike the robotic cars developed in the abovementioned research studies, not only the vehicle itself but also the camera's pan-tilt-position was controlled via SSVEP. The main task of this BCI study was to maneuver the MRC through a predetermined route and to examine the surroundings (live scenario task). The BCI performance was tested with 61 subjects. BCI key parameters, for example, stimulation frequencies, were determined individually by Wizard software. In this study, we tested the following hypotheses:

- Is it possible to use an SSVEP-based BCI stimulation and a background video on the same screen, without the negative influence of the presented video, as reported in, for example, [27, 30]?
- How does the video feedback affect BCI performance when simultaneously presented with the SSVEP-based BCI stimulation?
- Is our self-made, semiautonomous mobile robotic car suitable for future use as an assistive technology?
- How did the subjects perform using the presented SSVEP-based BCI control application, compared to results presented in, for example, [31]?

The paper is organized as follows: Section 2 describes the experimental setup and presents details about the experimental procedure and the hardware and software solutions. The results are presented in Section 3, followed by a discussion and conclusion in Sections 4 and 5, respectively.

## 2. Material and Methods

### 2.1. Subjects

Everyone who volunteered to participate in the study became a research subject after reading the subject information sheet. This study was carried out with written informed consent from all subjects in accordance with the Declaration of Helsinki. 61 subjects (30% female) with a mean (SD) age of 22.62 (5.00) years participated in the study, all healthy and all students or employees of the Rhine-Waal University of Applied Sciences in Kleve. The EEG recording took place in a normal PC-laboratory room (≈36 m^2^). The LCD screen was located in front of windows and the luminance was kept at an acceptable level, in order not to disturb the subjects. If necessary, outer blinds were pulled down during the day, or light was turned on during the evening. None of the subjects had neurological or visual disorders. Spectacles were worn when appropriate. Subjects did not receive any financial reward for their participation in this study.

### 2.2. Hardware

#### 2.2.1. Mobile Robotic Car (MRC)

**Figure 1:**
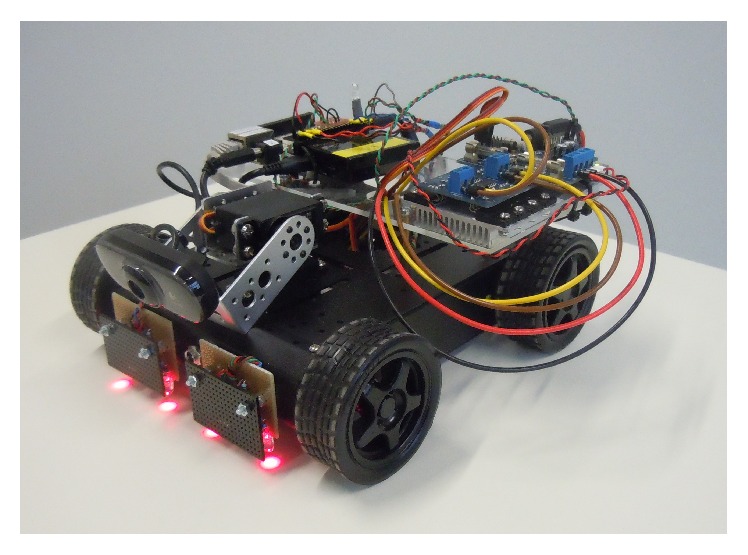
Mobile robotic car (MRC). On top of the MRC: the BeagleBone Board Rev A6, the Atmega168-based motor drive shield, and a small HAMA WiFi/WLAN-Access-Point. At the front: the red LEDs from light sensors that allowed the MRC to follow the white line on autopilot mode and the Logitech C210 camera mounted on 2 servos creating the pan-tilt head for 2-DoF (degree-of-freedom) control. The rechargeable battery is mounted inside the main case. MRC dimensions are 25 × 25 × 12 cm.

Four identically constructed mobile robotic cars (MRCs) were used in the experiment, all set up with the same hardware and software configurations. Four wheels were attached to a preassembled case (14 cm × 19 cm) with built-in DC motors. A rechargeable battery (6x AA NiMH, 1900 mAh) was placed inside. Four light sensors (each with a corresponding red LED, L-53SRC-F, for luminescence positioned side by side) were mounted on a prototype board at the front of the MRC. Two of those sensors were connected to the analog input pins of a BeagleBone Board Rev A6 at the center and two at the sides. The DC motors were controlled by an Atmega168-based unit. The camera, Logitech C210 (V-U0019), was mounted in front of the MRC on two servos (RB-421, RobotBase, China), simulating a pan-tilt head and connected to the BeagleBone USB port. The camera was controlled in five predefined orientations: looking down, forward, up, to the left, and to the right. The connection with the MRC was established through UDP via a HAMA N150 2in1-WLAN-ADAPTER, connected with an Ethernet cable to the BeagleBone of the MRC, and a USB WiFi adapter (D-Link, DWL-G122) connected to the desktop computer. All the electronic components (boards and HAMA N150 Access-Point) were mounted on top of the MRC. The BeagleBone was equipped with an 8 GB SD-card, running Ubuntu ARM (Linux distribution, version 12.04) with MJPG-Streamer installed (version 0.1, http://mjpg-streamer.sf.net) for video streaming. One of the used MRCs is shown in Figure 1.

#### 2.2.2. Data Acquisition and Feature Extraction

The subjects were seated in front of a 24′′ LCD screen (BenQ XL2420T or BenQ XL2411T, resolution: 1920 × 1080 pixels, vertical refresh rate: 120 Hz) at a distance of approximately 60 cm. The computer system based on an Intel Core i7, 3.40 GHz, was running Microsoft Windows 7 Enterprise. Standard passive Ag/AgCl electrodes were used to acquire the signals from the surface of the scalp. The ground electrode was placed over *AF*_*Z*_, the reference electrode was placed over *C*_*Z*_, and eight signal electrodes were placed at predefined locations on the EEG-cap marked with *P*_*Z*_, *PO*_3_, *PO*_4_, *O*_1_, *O*_2_, *O*_*Z*_, *O*_9_, and *O*_10_ according to the international system of EEG electrode placement. Standard abrasive electrolytic gel was applied between the electrodes and the scalp to bring impedances below 5 kΩ. An EEG amplifier g.USBamp (Guger Technologies, Graz, Austria) was utilized. The sampling frequency was set to 128 Hz. During the EEG signal acquisition, an analog bandpass filter between 2 and 30 Hz and a notch filter around 50 Hz were applied directly in the amplifier.

The minimum energy combination (MEC) method was originally introduced by Friman et al. in [40]. In this study, a refined version of the MEC, as presented by Volosyak in [18], was used. In the following, we present this method in a very short form.

#### 2.2.3. SSVEP Signal Model

We can model the signal recorded at a given electrode *i* (against reference electrode at time *t*) as follows:

(1) $$\begin{array}{c} y_{i}\left(\;t\;\right)=\overset{N_{h}}{\underset{k=1}{\sum }}\left(\;a_{i,k}\sin2\pi kft+b_{i,k}\cos2\pi kft\;\right)+E_{i,t}. \end{array}$$

The first part is the SSVEP evoked response consisting of sine and cosine functions of the frequency *f* and its harmonics *k*, with corresponding amplitudes *a*_*i*,*k*_ and *b*_*i*,*k*_. The last part *E*_*i*,*t*_ represents the noise component of the electrode *i*, other activities not related to the SSVEP evoked response, and artifacts. The *i*th EEG signals samples stored in *N*_*t*_ are in vector form *y*_*i*_ = *Xτ*_*i*_ + *E*_*i*_, where *y*_*i*_ = [*y*_*i*_(1),…, *y*_*i*_(*N*_*t*_)]^*T*^ is *N*_*t*_ × 1 vector. *X* is the SSVEP model matrix of size *N*_*t*_ × 2*N*_*h*_ and contains the sin (2*πkft*) and cos (2*πkft*) pair in its columns. The *τ*_*i*_ vector contains the corresponding amplitudes. This is based on the assumption that the nuisance is stationary within the short time segment length; the signals from the *N*_*y*_ electrodes can be stored in a vector form *Y* = [*y*_1_,…, *y*_*N*_*y*__].

#### 2.2.4. Minimum Energy Combination

In order to cancel out the nuisance and noise and to boost the SSVEP response, channels are created in a weighted sum of *N*_*y*_ electrodes *s* = ∑_*i*=1_^*N*_*y*_^ *w*_*i*_ *y*_*i*_ = *Yw*. Several sets *S* of *N*_*s*_ channels can be created with a different combination of the original electrode signals *S* = *YW*, where *W* is *N*_*y*_ × *N*_*s*_ matrix that contains the different weight combinations. The structure of *W* is based on the minimum energy combination technique. In order to use this combination method for cancelling the nuisance signal, in a first step, any potential SSVEP component needs to be removed from the signal: $\tilde{Y}=Y-X(X^{T}X{)}^{-1}X^{T}Y$. The next step is to find a weight vector $\hat{w}$ that minimizes the resulting energy of the combination of the electrodes' noise signals $\tilde{Y}$: $\tilde{Y}\hat{w}$. The last step is the optimization problem for minimalizing the nuisance and noise component:

(2) $$\begin{array}{c} \underset{\hat{w}}{min}{\left\|\;\tilde{Y}\hat{w}\;\right\|}^{2}=\underset{\hat{w}}{min}{\;\hat{w}}^{T}{\tilde{Y}}^{T}\tilde{Y}\hat{w}. \end{array}$$

The solution to the minimize problem is the smallest eigenvector *v*_1_ (of the symmetric matrix ${\tilde{Y}}^{T}\tilde{Y}$) and the energy of the resulting combination equals the smallest eigenvalue *λ*_1_ from this matrix. In order to discard up to 90% of the nuisance signal, the total number of channels is selected by finding the smallest value for *N*_*s*_ that satisfies the following equation:

(3) $$\begin{array}{c} \frac{\sum_{i=1}^{N_{s}}\lambda_{i}}{\sum_{j=1}^{N_{y}}\lambda_{j}}>0.1. \end{array}$$

To detect the SSVEP response for a frequency, the power of that frequency and its harmonics *N*_*h*_ is predicted:

(4) $$\begin{array}{c} \hat{P}=\frac{1}{N_{s}N_{h}}\overset{N_{s}}{\underset{l=1}{\sum }}\;\overset{N_{h}}{\underset{k=1}{\sum }}{\left\|\;X_{k}^{T}s_{l}\;\right\|}^{2}. \end{array}$$

To prevent overlapping between frequencies, we use *N*_*h*_ = 2 in the actual system implementation. The estimation of the power of the SSVEP stimulation frequencies, and additional frequencies (*N*_*f*_), is normalized into probabilities:

(5) $$\begin{array}{c} p_{i}=\frac{{\hat{P}}_{i}}{\sum_{j=1}^{j=N_{f}}{\hat{P}}_{j}}\;with\;\;\overset{i=N_{f}}{\underset{i=1}{\sum }}p_{i}=1, \end{array}$$

where ${\hat{P}}_{i}$ is the power estimation of the *i*th signal, 1 ≤ *i* ≤ *N*_*f*_. To increase the difference between the results, we apply a Softmax function:

(6) $$\begin{array}{c} p_{i}^{\prime }=\frac{e^{\alpha p_{i}}}{\sum_{j=1}^{j=N_{f}}e^{\alpha p_{j}}}\;with\;\;\overset{i=N_{f}}{\underset{i=1}{\sum }}p_{i}^{\prime }=1, \end{array}$$

where *α* is set to 0.25. The output values of the Softmax function are between 0 and 1 and their sum is equal to 1. The classification output *C*_*o*_ of the signal of *i*th frequency is a result of three conditions: the probability *p*_*i*_′ of the *i*th frequency is the highest, it surpasses a predefined border *p*_*i*_′ ≥ *β*_*i*_, and the detected frequency is one of the stimulation frequencies. After each classification, to prevent wrong classifications, the classifier output was rejected for the next 9 blocks (approximately 914 ms of EEG data with the sampling rate of 128 Hz), and the visual stimulation was paused for that time period. This classification method is the next iteration of the Bremen-BCI as presented, for example, in [18]. An example of the recorded signal and the SNR can be seen in Figure 9.

**Figure 2:**
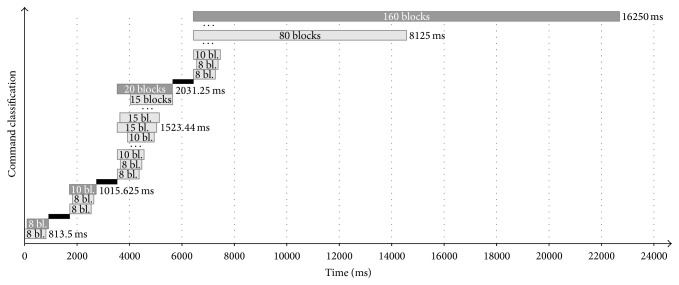
Changes in the time segment length after a performed classification in case no distinct classification can be made and the actual time *t* allows for the extension to the next predefined value. After each classification (gray bars of 8, 10 20, and 160 blocks), additional time for gaze shifting was included (black) in which the classifier output was rejected for 9 blocks.

The SSVEP classification was performed online every 13 samples (ca. 100 ms) on the basis of the adaptive time segment length of the acquired EEG data introduced in [18]. If no classification was possible, the time segment length window was gradually extended to the next predefined window lengths (see Table 1 and Figure 2).

### 2.3. Software

#### 2.3.1. Graphical User Interface

**Figure 3:**
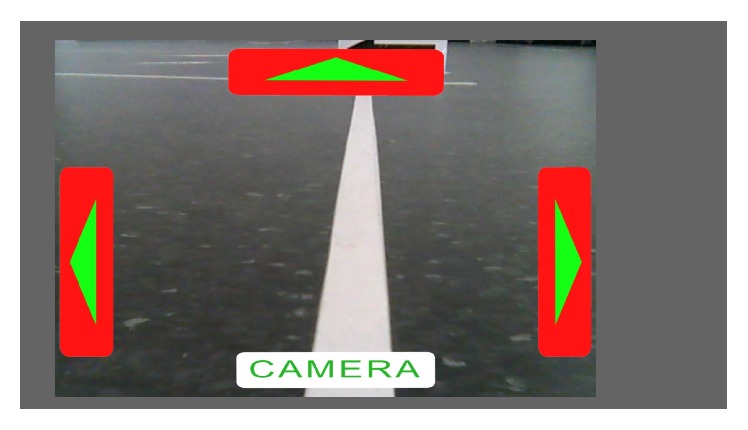
A screenshot of the* robot-GUI* in drive mode. In this situation, the user had to select the “drive forward” command, which caused the robot to execute an autopilot program that uses the built-in light sensors to follow the white line.

**Figure 4:**
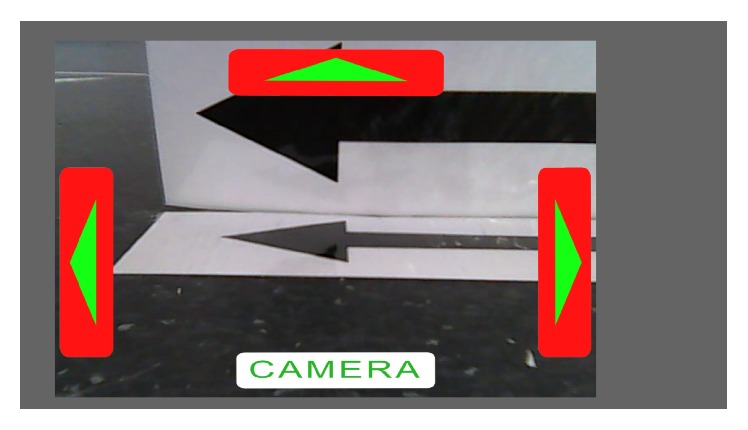
Signs, positioned at the turning points and positioned towards the camera, showed the correct direction for following the track. Here in the screenshot, the robot automatically stopped after reaching this turning point and a sign instructed the user to select the command “turn left.”

A custom-made graphical user interface (*robot-GUI*) was designed to control the MRC. DirectX 9 was used to draw the images (buttons and a real-time picture from the actual camera view in background) on the monitor, and OpenCV library (version 2.4.10, http://www.opencv.org/) was used for image acquisition. Each received frame was rendered into background texture as soon as it was received (max. 30 Hz; the frame rate was limited by the used camera). Each received frame (640 × 480 pixels) was rescaled to 1280 × 960. Due to the ratio of the streamed video frames and the resolution of the monitors used, a gray background in the form of a frame was implemented to prevent unnatural video scaling. The starting dimensions of the buttons were 146 × 512 pixels for left and right, 576 × 128 pixels for the top, and 536 × 96 pixels for bottom buttons (see Figures 3 and 4). During the runtime, the size of the buttons changed continuously, depending on the calculated probabilities of the *i*th stimuli in (6). The* robot-GUI* had two modes: a drive mode and a camera mode. In drive mode, the subject was able to send the following commands to the MRC: “drive forward” (a discrete command, using the line-sensors to follow the taped line on autopilot to the next direction change), “turn left,” “turn right,” and “switch to camera mode.” In camera mode, the subject was able to send the following commands: “look left,” “look right,” “look up,” and “switch to drive mode.” While looking up, the button “look up” changed the content and functionality to “look forward.” The control buttons were red in drive mode and yellow in camera mode to increase user friendliness. The aspect ratio of the video stream was 4 : 3, so the computer screen was not entirely filled out. If the command “drive forward” was selected by the user, an autonomous program was executed on the MRC. The MRC used the four line-sensors to follow the white line taped to the floor until it arrived at a turning point, where the robot stopped automatically. While driving, the MRC automatically made small corrections when the line was detected by one of the inner sensors; the rotation of the wheels on the same side was decreased by 50% until another sensor detected the line. Similarly, when the line was detected by one of the outer sensors, the wheels on the same side were stopped so that the MRC could drive in a curve, in order to go back on track. If one of the turning commands was selected, the MRC made an approximately 90-degree turn in the corresponding direction. In drive mode, the camera was facing slightly the ground (approximately 20 degrees down from the frontal view for the “look down” position). When switching to camera mode (represented by the word “CAMERA” at the bottom of the* robot-GUI*), the camera changed to the “look forward” position (switching back to drive mode would change the camera position to looking down again). All the EEG data and the received video stream from the MRC were anonymously stored.

#### 2.3.2. Wizard

The Wizard software is, generally spoken, a calibration program that autonomously determines BCI key parameters [37]. It is designed to select user-specific optimal stimulation frequencies for SSVEP-based BCIs on LCD screens. It is a development of the method presented in [41]. The Wizard chooses four frequencies with the best SSVEP response out of a set of 14 possible stimulation frequencies *f*_*i*_ (ranged from 6 Hz to 20 Hz) that can be realized on a monitor screen with a vertical refresh rate of 120 Hz [42]. In the following, we provide a brief description.

**Step 1 (alpha test).**

The subject was instructed by an audio feedback to close his or her eyes. 15 frequencies in the alpha wave band (8.27, 8.57, 8.87, 8.93, 9.23, 9.53, 9.70, 10.0, 10.30, 10.61, 10.91, 11.21, 11.70, 12.0, and 12.30 Hz) were measured for 10 seconds during the closed eyes period and if any of those 15 frequencies would interfere with a possible stimulation frequency *f*_*i*_ (Δ*f* ≤ 0.15 Hz), this frequency *f*_*i*_ would be filtered out.

**Step 2 (stimulation frequency selection).**

**Figure 5:**
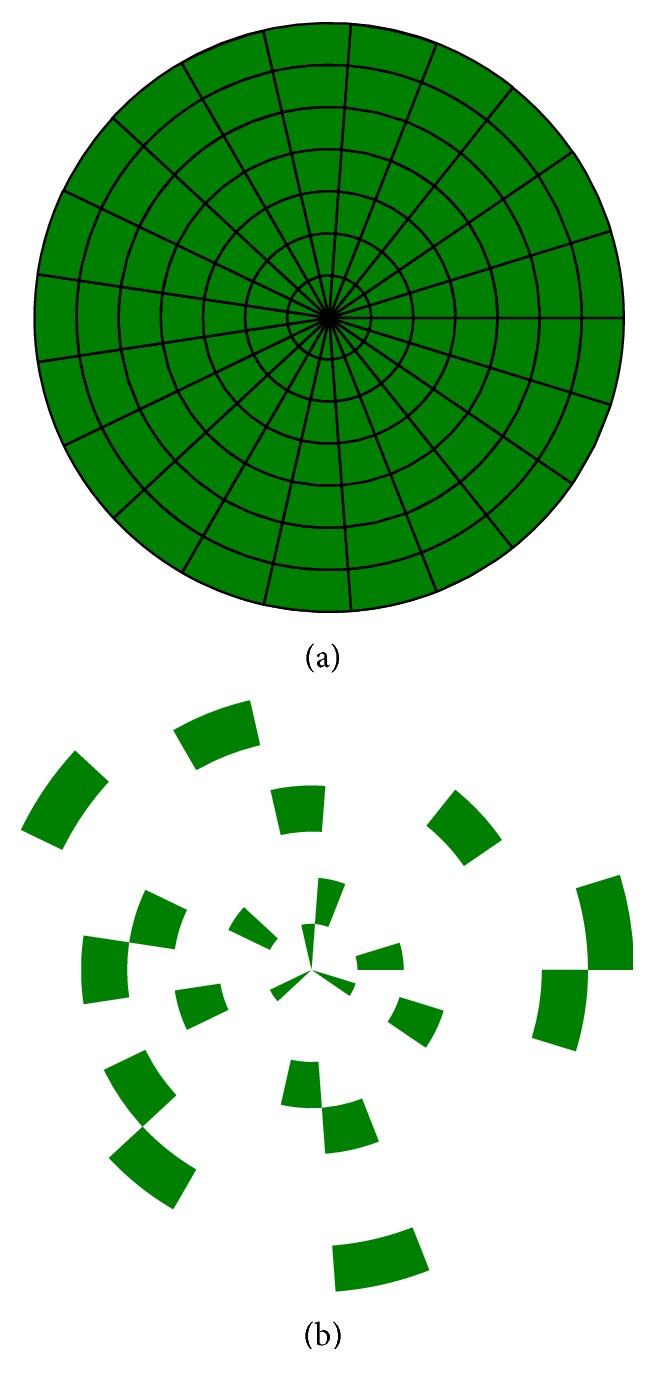
(a) shows the second step of the Wizard. The subject faced a circle consisting of small segments which flickered simultaneously with seven different frequencies. Each of the seven tested frequencies was represented by the same amount of segments. The segments representing one of the seven tested frequencies were scattered in random order (b) forming the stimulation circle (a). After this multistimulation with thirteen or fourteen different frequencies, depending on the alpha test result, the EEG data were analyzed and ordered from the strongest to the lowest SSVEP response.

In order to avoid the influence of harmonic frequencies, the user had to look at two circles in sequence. Each circle contained seven stimulation frequencies which were presented in pseudorandom order as small, green circle segments (see Figure 5). This step took 20 seconds.

**Step 3 (optimal classification thresholds and time segment length).**

**Figure 6:**
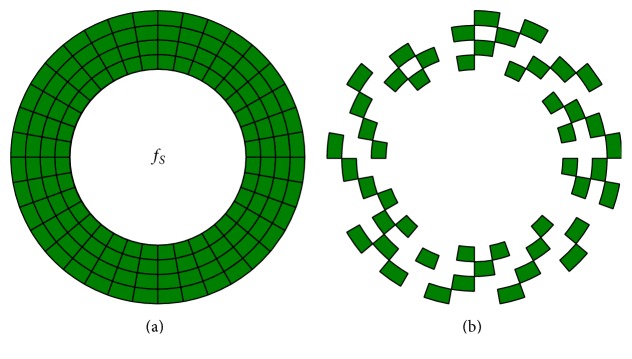
This figure shows the third step of the Wizard (a). The main frequency *f*_*S*_ is in the center (white circle stimulation) and the other three frequencies (from four *f*_*S*_*i*__ with the strongest SSVEP response) are divided into small stimulation blocks and scattered around *f*_*S*_ acting as “noise” in peripheral vision. This pattern is shown in (b). This was repeated for each *f*_*S*_*i*__.

In this step, the Wizard software chose the best starting time-length segment *T*_*S*_ as well as the optimal classification thresholds *β*_*i*_ for the frequencies determined in the previous step. First, a white circle, flickering at the frequency which had the highest SSVEP response in phase 2, was displayed. It was necessary to simulate noise caused by peripheral vision when concentrating on the target object. For this purpose, the white circle was surrounded by a green ring, containing segments which presented the remaining optimal frequencies (see Figure 6). The user was instructed by an audio feedback to gaze at the white circle. The circle and the ring flickered for 10 seconds while EEG data were recorded. After a two-second break, the flickering continued. The white circle now flickered with the second-highest frequency from phase two, while the ring flickered with the remaining three frequencies. This procedure was repeated until the data for all four optimal frequencies were collected. Total recording time for phase three was 40 seconds, and based on this data, optimal thresholds and time segment length were determined. If one of the frequencies *f*_*S*_*i*__ did not reach a defined, minimal threshold *β*_*i*_, this frequency was replaced with the next best frequency from Step 2, and the procedure of this step was repeated.

### 2.4. Procedure

**Figure 7:**
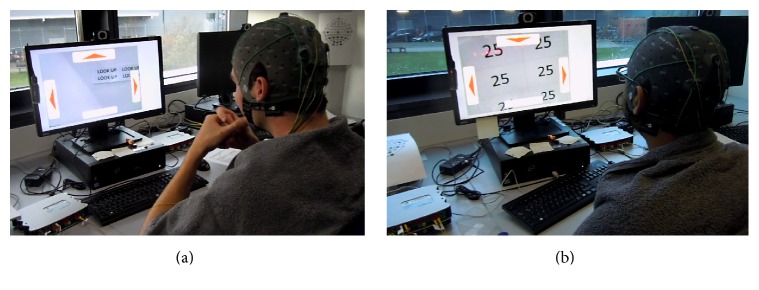
Two subjects during the driving task. The* robotic-GUI* is in camera mode. The subject in (a) sees a text message instructing him to select the button “look up.” The subject in (b) is one step further and is facing the hidden secret number which is attached underneath a drawer.

**Figure 8:**
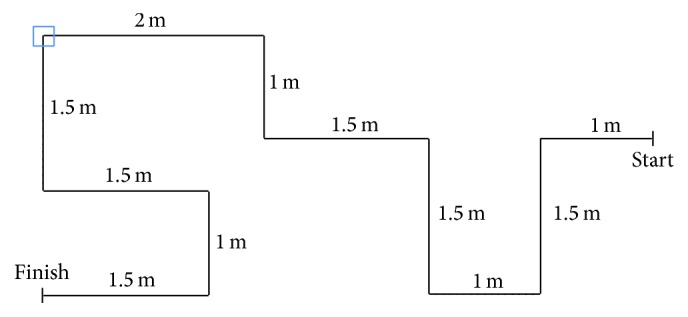
Track for the mobile robotic car. Subjects had to steer the MRC through a 15-meter-long course with 10 turns in order to reach the finish line. At a certain turning point (marked with a blue square), the MRCs camera faced a text message that instructed the subject to switch to camera mode. The task was to examine the surroundings and to look for a hidden number which was attached underneath a drawer (otherwise not visible). One example of the optimal path is shown in Figure 9.

In order to analyze the BCI performance variety, a pre- and postquestionnaire system (similar to [21]) was employed. After signing the consent form, each subject completed a brief prequestionnaire and was prepared for the EEG recording. Subjects were seated in a comfortable chair in front of the monitor, the electrode cap was placed, and electrode gel was applied. First, the subjects went through the steps of the Wizard and BCI key parameters (stimulation frequencies, classification thresholds, and optimal time segment length) were determined. Then, the experimenter started the* robot-GUI* (Figure 3). The control task was to drive the MRC through a predetermined route (Figure 8). If the* robot-GUI* software recognized a wrong command, for example, “turn right” instead of “turn left,” this command was not sent, so that the MRC would not drive off the track (Figure 7). If the subject made a few mistakes at the beginning and was willing to start over again (this occurred with a small number of subjects), the experimenter moved the MRC back to the starting point and restarted the* robot-GUI*. In the very few cases where the wireless connection to the MRC was interrupted (e.g., the MRC was mechanically unable to execute the command that was correctly selected in the robot-GUI by the user), the experimenter manually repositioned the MRC to the next turning point.

At a certain turning point (represented by a blue square shown in Figure 8, when approximately 60% of the driving task was completed), the camera was facing a text message, which instructed the subjects to look up (user action represented with a circled number as, e.g., ⑭ in this case). At this point, the user had to switch to camera mode and select the command “look up.” Now the subject was able to see a hidden secret number which was attached to the bottom of a drawer (located at a height of 50 cm, facing the floor, not normally visible to the people in the room). The numbers varied randomly between the subjects and were only accessible via the MRC camera. After seeing the number on the screen, the subject had to change back to drive mode and continued the driving task. When arriving at the finish line, the camera faced another text message which again instructed the subjects to look up. After changing to camera mode and selecting “look up,” the camera faced a sign displaying the word “finish” and the* robot-GUI* stopped automatically. All of the EEG data and performed commands were stored on the hard drive of the desktop PC for further evaluation. The values of ITR and accuracies were calculated automatically by the software. The ITR was calculated using the formula presented by Wolpaw et al. in [43]; the number of possible choices for classification was equal to four; it was the same for each mode (drive mode or camera mode). After finishing the experiment, the subject filled out the postquestionnaire.

## 3. Results

The results of the driving performance are shown in Table 3. All 61 subjects were able to control the BCI application and completed the entire driving task. The overall mean (SD) ITR of the driving performance was 14.07 (4.44) bits/min. The mean (SD) accuracy was 97.14 (3.73)%. Except for one, all subjects reached accuracies above 80%. 40 subjects reached accuracies above 90%, and nineteen of them even completed the steering task without any errors. The mean (SD) accuracy of each action (commands required to complete the task) was 96.14 (0.02)%. In the prequestionnaire, subjects answered the questions regarding gender, the need for vision correction, tiredness, and BCI experience, as displayed in Table 2. Eighteen female subjects (30%) with an average (SD) age of 22.67 (4.30) years and 43 male subjects with an average (SD) age of 22.60 (5.32) years participated in the experiment. The influence of gender was also tested, 18 female subjects achieved a mean (SD) accuracy and ITR of 93.24 (7.98)% and 14.05 (5.73) bits/min, respectively, and 43 male achieved a mean (SD) accuracy and ITR of 92.95 (6.59)% and 14.07 (5.08) bits/min, respectively. A one-way ANOVA showed no statistically significant difference in accuracy *F*(1,59) = 0.022, *p* = 0.883, and ITR *F*(1,59) = 0.0003, *p* = 0.987, between those two groups. The influence of vision correction on accuracy and ITR was also tested. A total of 22 subjects required vision correction in the form of glasses or contact lenses (as they stated in the questionnaire). They achieved a mean (SD) accuracy and ITR of 91.56 (6.36)% and 12.78 (4.02) bits/min, respectively. These results were compared to subjects who did not require vision correction and achieved a mean (SD) accuracy and ITR of 93.92 (5.35)% and 14.84 (4.66) bits/min, respectively. A one-way ANOVA showed no statistically significant influence of vision correction on accuracy *F*(1,59) = 1.664, *p* = 0.202. There was also no significant effect of vision correction on ITR *F*(1,59) = 2.263, *p* = 0.138. An evaluation of tiredness was also calculated on the basis of the answers given in a prequestionnaire (scale from 1 to 5). Results of the prequestionnaire are shown in Table 2. A one-way ANOVA revealed no significant effect of tiredness on accuracy *F*(4,56) = 0.335, *p* = 0.853, and ITR *F*(4,56) = 0.067, *p* = 0.991.

## 4. Discussion

In the results presented, the overall accuracy was calculated for all commands in drive mode and in camera mode without distinguishing between them, since four commands for SSVEP classification were always present. Even though different commands were sent, the command classification remained the same. A comparison of this study to other publications on steering a robot with SSVEP-based BCI is presented in Table 4.

In order to test the influence of video overlay, we compared the accuracy results with a demographic study [21] in which 86 subjects achieved a mean (SD) accuracy of 92.26 (7.82)% while driving a small robot through a labyrinth with a four-class SSVEP-based BCI. An unpaired *t*-test showed no statistically significant difference *t*(145) = −0.089, *p* = 0.397, compared to the present values. This shows that the video overlay did not negatively influence the BCI performance. Gergondet et al. listed three factors with respect to how the live video feedback in the background can influence the SSVEP stimulation: distraction due to what is happening on the video, distraction due to what is happening around the user, and variation in luminosity below the stimuli shown to the user [30]. Kapeller et al. showed that there is a slight decrease in performance for the underlying video sequence due to a distractive environment and small targets used [27]. Contrary to the findings reported by Gergondet et al. and Kapeller et al., the dynamic background did not noticeably affect the BCI performance (unpaired *t*-test result). We assume that this was due to the fact that stimulation frequencies and minimal time segment classification windows were adjusted individually and that relatively large stimulation boxes were used (visual angle).

The high accuracies of the SSVEP-based BCI system support the hypothesis that BCI can be used to control a complex driving application. In comparison to the study by Diez et al. [31], the MRC self-driving behavior was limited to a preset number of turning points and a predefined path. Although the robot presented by Diez et al. could avoid obstacles by itself (limited algorithm reaction), it decreased its speed for trajectory adjustment and, in some cases, stopped to avoid collision, and further user commands were necessary. While Diez et al. reported a mean time of 233.20 seconds for the second destination (13.9-meter distance), in this study the achieved mean time was 207.08 seconds (15-meter distance). As pointed out by Diez et al., the overall time for completing the task depends not only on the robot but also on the subject's performance controlling the BCI system [31]. In our study, the high accuracies of the BCI system were achieved due to the key parameter adjustment carried out by the Wizard software.

The main difference between the robot used by Diez et al. and the MRC presented in this study is the extended control possibility of the interface that also allows for controlling the camera. Both robots were designed for different purposes: Diez et al. mainly designed their robot for driving from point A to point B without any additional interaction with the environment, as they planned to use the system for wheelchair control in the future. By contrast, in our design, the MRC itself is the interaction device. Equipped with a display and a two-way audio system, it might serve as a remote telepresence in the future.

The SSVEP paradigm allows for a robust implementation of four driving commands. Compared to robots constructed for the P300 BCI approach, where the user faces large stimulation matrices spread across the entire screen, the number of simultaneously displayed targets here is only four, and hence the load on the visual channel is considerably lower in comparison. Therefore, the user might find it easier to concentrate on the driving task. On the other hand, the lesser number of command options reduces freedom and speed. However, a shared-control paradigm allows for complex tasks to be executed with minimal effort. The command “drive forward” starts an autopilot program that guides the robot through a straight distance until it reaches a predefined stopping point.

**Figure 9:**
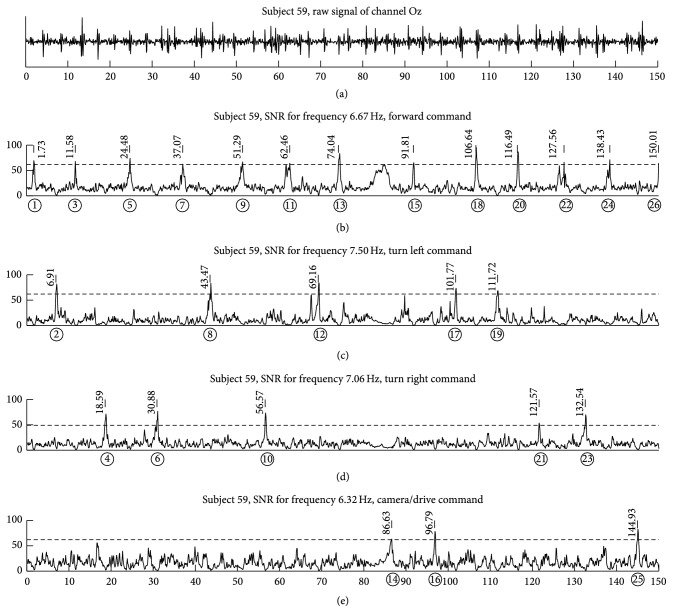
This chart presents exemplarily the BCI performance for subject 59 (optimal path with 100% accuracy). The top row shows the raw EEG signal acquired from channel *O*_*z*_ (one of eight EEG electrodes). The remaining graphs show the SNR values during the whole experiment (dashed line represents the threshold for each command) for the four commands forward/up, turn left, turn right, and camera/drive; the stimulation frequencies for those commands were 6.67, 7.50, 7.06, and 6.32 Hz, respectively. The *x*-axis, the same for all graphs, represents the total duration of the experiment (150 seconds). The marking above each graph represents the exact time of each classification; circled numbers below the *x*-axis correspond to the task actions.

**Figure 10:**
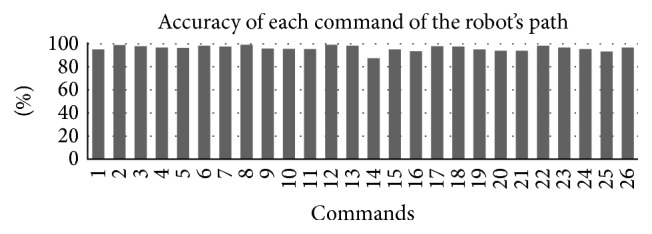
This chart presents the accuracy of each command along the path (*x*-axis) and its selection accuracy to previous correct selection over all subjects.

While the MRC executed the autopilot program and followed the line for some time, subjects purposely ignored the flickering targets and concentrated on the video feedback instead. As these waiting periods were added up to the actual classification times to each command that followed an autopilot command (“forward”), the ITR values are considerably low. From the researcher's point of view, a number of errors could have been avoided if some subjects would have paid more attention to the live feedback. Especially in the task of reading the hidden number, subjects were trying to drive along the path without looking at the text message that appeared on the live feedback. After realizing that the robot was not turning, they looked at the video and saw the text message instructing them to look up. This is visible in the analysis of the accuracy of the robots commands (see Figure 10). The lowest accuracy of 87.49% was for action “change view to the camera mode” (robot command 14); this may be caused by the fact that the subjects got used to the actions “turn left”/“turn right” and “drive forward” which they repeated six times from the starting point (see Figures 10 and 9). This could easily be avoided if the driving paths were designed with evenly distributed tasks (e.g., live scenario tasks, such as reading a hidden number, should occur more often).

No difference between the time needed for the selection of the command “drive forward” in the beginning of the experiment in comparison to the end of the experiment can be observed (see Table 5); hence, neither fatigue nor practice seemed to influence performance.

The general literacy rate among test subjects of 100% indicates that SSVEP-based BCIs can be used by a larger population than previously assumed. This high literacy is due to the careful selection of individual SSVEP key parameters, which were determined by the presented calibration software.

### 4.1. Limitations

The SSVEP-control application has some restrictions:

- The wireless connection was implemented with the UDP protocol and some information might therefore not be received properly.
- In order to maximize performance accuracy and user friendliness, the number of simultaneously displayed targets was restricted to four, which resulted in a considerably lower ITR.
- It should also be noted that, for long term use, recalibration might be inevitable. Although the number of targets is limited to four, the SSVEP-based BCI presented here depends on the user's vision and control over his or her eye movements. It therefore has to compete with other approaches such as P300 and other eye tracking systems. Visual stimulation with low frequencies in particular is known to cause fatigue.
- Moreover, subjects in this study may not be representative of the general population; they tended to be young healthy men. Further tests with older and physically impaired persons are required.

## 5. Conclusions

In the present study, the SSVEP-based driving application (*robot-GUI*) was proven to be successful and robust. The driving application with live video feedback was tested with 61 healthy subjects. All participants successfully navigated a mobile semiautonomous robot through a predetermined 15-meter-long route. BCI key parameters were determined by the Wizard software. No negative influence of the live video feedback on the performance was observed. Such applications could be used to control assistive robots but might also be of interest for healthy subjects. In contrast to P300, the SSVEP stimulation has to be sufficiently strong (in terms of size or visual angle) to significantly influence the user. A common problem of all BCI studies that also occurred here is to keep the user motivated and focused on the task. The evaluation of the questionnaire shows that for the majority of subjects the driving task with the SSVEP-based BCI was not fatiguing, even though lower stimulation frequencies, which usually cause more fatigue than higher frequencies, were used in the experiment. More specifically, frequencies between 6 and 12 Hz were determined in the calibration procedure, as they elicited the strongest SSVEP responses. Some subjects stated that as they concentrated more on the camera video, they did not find the flickering annoying at all. Our results prove that SSVEP-based, personalized, adaptive BCI systems with a low target number can be used as an everyday assistive technology for remote robots. We have also shown that it is possible to design a four-target interface with extended capabilities such as camera and driving control. The control application presented here can be used for mobile robots, as an assistive remote technology, as a game interface, for wheelchair control, or even for steering remotely controlled drones. This study should be considered as a step towards resolving the problems of semiautonomous mobile robots controlled with SSVEP-based BCIs. In order to further improve this approach, the next steps should be an online parameter adaptation mechanism for improving the accuracy through practicing, a possibility of turning the stimulation on/off, and a two-way video and audio communication for a truly remote telepresence.

## Figures and Tables

**Table 1:** Overview of the time windows *T*_*S*_ of EEG data used for SSVEP classification. Eleven time segments between 812.5 ms (8 × 13 samples or 8 blocks of EEG data) and 16250 ms were used in this study.

Time [ms]	Blocks of EEG data
812.5	8
1015.6	10
1523.4	15
2031.3	20
3046.9	30
4062.5	40
5078.1	50
6093.8	60
7109.4	70
8125	80
16250	160

**Table 2:** Questionnaire results. The table presents the answers collected from the prequestionnaire. The figures show the number of respondents or the mean (SD) value.

Age	Gender		Vision correction		Tiredness					Length of sleep last night	Experience with BCIs	
Years	Male	Female	Yes	No	1, not tired	2, little tired	3, moderately tired	4, tired	5, very tired	Hours	Yes	No
22.62 (5.00)	43	18	22	39	18	23	17	3	0	6.75 (1.22)	6	55

**Table 3:** Results of the driving performance. Total time each subject required for steering the MRC through the route (15 meters and 10 turns including camera movements). 26 correct commands (3 commands in camera mode and 23 commands in drive mode) were necessary to complete the task. Accuracy (Acc) is the number of correct commands (26) divided by the total number of commands (*C*_*n*_). Mean values are given at the bottom of the table.

Subject	Time	Acc	ITR	*C*_*n*_
	[s]	[%]	[bits/min]	
1	305.60	86.67	7.20	30
2	283.46	89.66	8.32	29
3	284.07	81.25	6.80	32
4	280.11	81.25	6.90	32
5	252.59	81.25	7.65	32
6	190.02	92.86	13.40	28
7	322.06	96.30	8.62	27
8	310.07	70.27	4.66	37
9	158.13	92.86	16.10	28
10	371.82	89.66	6.35	29
11	241.72	86.67	9.10	30
12	151.33	92.86	16.83	28
13	155.49	100.00	20.07	26
14	210.54	96.30	13.18	27
15	208.41	100.00	14.97	26
16	241.62	83.87	8.52	31
17	241.72	86.67	9.10	30
18	145.13	96.30	19.12	27
19	167.48	96.30	16.57	27
20	220.80	92.86	11.53	28
21	194.19	96.30	14.29	27
22	431.54	92.86	5.90	28
23	163.52	100.00	19.08	26
24	171.84	86.67	12.80	30
25	248.12	83.87	8.30	31
26	131.93	100.00	23.65	26
27	161.38	100.00	19.33	26
28	184.75	96.30	15.02	27
29	144.22	100.00	21.63	26
30	167.48	92.86	15.20	28
31	216.63	92.86	11.75	28
32	355.16	81.25	5.44	32
33	244.06	86.67	9.01	30
34	152.14	100.00	20.51	26
35	195.61	96.30	14.18	27
36	239.48	83.87	8.60	31
37	166.36	89.66	14.18	29
38	172.45	100.00	18.09	26
39	218.97	83.87	9.40	31
40	165.65	89.66	14.25	29
41	146.66	100.00	21.27	26
42	186.27	89.66	12.67	29
43	221.31	92.86	11.51	28
44	259.29	86.67	8.48	30
45	157.02	100.00	19.87	26
46	147.88	96.30	18.76	27
47	187.18	96.30	14.82	27
48	145.54	100.00	21.44	26
49	160.57	96.30	17.28	27
50	204.95	100.00	15.22	26
51	228.11	96.30	12.16	27
52	160.37	100.00	19.46	26
53	236.64	81.25	8.17	32
54	148.89	100.00	20.95	26
55	150.11	100.00	20.78	26
56	139.85	100.00	22.31	26
57	147.16	100.00	21.20	26
58	242.73	100.00	12.85	26
59	150.01	100.00	20.80	26
60	164.63	96.30	16.85	27
61	178.85	96.30	15.51	27

Mean	* 207.08 *	* 93.03 *	* 14.07 *	*28.11 *
SD	50.25	5.73	4.44	1.82
Min.	131.93	70.27	4.66	26.00
Max.	431.54	100.00	23.65	37.00

**Table 4:** Comparison of BCI systems for robot navigation.

Publication	Subjects	Classes	Literacy rate [%]	Mean accuracy [%]	Mean ITR [bits/min]	Robot type
Volosyak et al. [21]	86	4	98	92.26	17.24	E-puck
Zhao et al.^1^ [34]	7	4	100	90.3	24.7	NAO
Kapeller et al.^2^ [27]	11	4	91	91.36	N/A	E-puck
Diez et al.^3^ [31]	7	4	100	88.80^*∗*^	N/A	Pioneer 3-DX
Holewa and Nawrocka [24]	3	4	100	73.75	11.36	Lego Mindstorm
Choi and Jo^1^ [32]	5	2	100	84.4	11.40	NAO
This study	61	4	100	93.03	14.07	MRC

^1^Results for SSVEP paradigm.

^2^f-VEP results for 4 classes, without zero class.

^3^Results for destination 1, ^*∗*^correct/(correct + wrong) commands.

**Table 5:** Distribution of the time blocks needed for selection of the first three commands “drive forward” and the last three commands “forward”/“look up” over all subjects.

Distribution [%]								
Time windows *T*_*S*_ in EEG data blocks								
	8	10	15	20	30	40	50	60 to 160
①	6.56	18.03	18.03	22.95	11.48	6.56	8.20	8.20
③	8.20	22.95	8.20	19.67	11.48	8.20	4.92	16.39
⑤	9.84	13.11	8.20	19.67	6.56	9.84	8.20	24.59

Mean	* 8.20 *	* 18.03 *	* 11.48 *	* 20.77 *	* 9.84 *	* 8.20 *	* 7.10 *	* 16.39 *

㉒	9.84	18.03	14.75	18.03	9.84	3.28	6.56	19.67
㉔	11.48	16.39	16.39	14.75	13.11	9.84	1.64	16.39
㉖	11.48	14.75	11.48	22.95	6.56	6.56	4.92	21.31

Mean	* 10.93 *	* 16.39 *	* 14.21 *	* 18.58 *	* 9.84 *	* 6.56 *	* 4.37 *	* 19.13 *

## References

[1] Cheney M., Uth R. (1999). *Tesla: Master of Lightning*.

[2] Walter W. (1953). *The Living Brain*.

[3] Flandorfer P. (2012). Population ageing and socially assistive robots for elderly persons: the importance of sociodemographic factors for user acceptance. *International Journal of Population Research*.

[4] Nijholt A., Tan D., Pfurtscheller G. (2008). Brain-computer interfacing for intelligent systems. *IEEE Intelligent Systems*.

[5] Millán J. D. R., Rupp R., Müller-Putz G. R. (2010). Combining brain-computer interfaces and assistive technologies: state-of-the-art and challenges. *Frontiers in Neuroscience*.

[6] Brunner C., Birbaumer N., Blankertz B. (2015). BNCI horizon 2020: towards a roadmap for the BCI community. *Brain-Computer Interfaces*.

[7] Bogue R. (2009). Exoskeletons and robotic prosthetics: a review of recent developments. *Industrial Robot*.

[8] Ron-Angevin R., Velasco-Alvarez F., Sancha-Ros S., da Silva-Sauer L. (2011). A two-class self-paced BCI to control a robot in four directions. *IEEE International Conference on Rehabilitation Robotics (ICORR '11)*.

[9] Tonin L., Carlson T., Leeb R., Millán J. D. R. Brain-controlled telepresence robot by motor-disabled people.

[10] Huang D., Qian K., Fei D.-Y., Jia W., Chen X., Bai O. (2012). Electroencephalography (EEG)-based brain-computer interface (BCI): a 2-D virtual wheelchair control based on event-related desynchronization/synchronization and state control. *IEEE Transactions on Neural Systems and Rehabilitation Engineering*.

[11] Chella A., Pagello E., Menegatti E. A BCI teleoperated museum robotic guide.

[12] Escolano C., Antelis J. M., Minguez J. (2012). A telepresence mobile robot controlled with a noninvasive brain-computer interface. *IEEE Transactions on Systems, Man, and Cybernetics, Part B: Cybernetics*.

[13] Vialatte F.-B., Maurice M., Dauwels J., Cichocki A. (2010). Steady-state visually evoked potentials: focus on essential paradigms and future perspectives. *Progress in Neurobiology*.

[14] Regan D. (1966). Some characteristics of average steady-state and transient responses evoked by modulated light. *Electroencephalography and Clinical Neurophysiology*.

[15] Frank Y., Torres F. (1979). Visual evoked potentials in the evaluation of ‘cortical blindness’ in children. *Annals of Neurology*.

[16] Ian McDonald W., Compston A., Edan G. (2001). Recommended diagnostic criteria for multiple sclerosis: guidelines from the international panel on the diagnosis of multiple sclerosis. *Annals of Neurology*.

[17] Gao S., Wang Y., Gao X., Hong B. (2014). Visual and auditory brain-computer interfaces. *IEEE Transactions on Biomedical Engineering*.

[18] Volosyak I. (2011). SSVEP-based Bremen-BCI interface—boosting information transfer rates. *Journal of Neural Engineering*.

[19] Müller-Putz G. R., Pfurtscheller G. (2008). Control of an electrical prosthesis with an SSVEP-based BCI. *IEEE Transactions on Biomedical Engineering*.

[20] Martinez P., Bakardjian H., Cichocki A. (2007). Fully online multicommand brain-computer interface with visual neurofeedback using SSVEP paradigm. *Computational Intelligence and Neuroscience*.

[21] Volosyak I., Valbuena D., Lüth T., Malechka T., Gräser A. (2011). BCI demographics II: how many (and What Kinds of) people can use a high-frequency SSVEP BCI?. *IEEE Transactions on Neural Systems and Rehabilitation Engineering*.

[22] Zhao J., Meng Q., Li W., Li M., Chen G. SSVEP-based hierarchical architecture for control of a humanoid robot with mind.

[23] Guneysu A., Levent Akin H. An SSVEP based BCI to control a humanoid robot by using portable EEG device.

[24] Holewa K., Nawrocka A. Emotiv EPOC neuroheadset in brain—computer interface.

[25] Kwak N.-S., Müller K.-R., Lee S.-W. Toward exoskeleton control based on steady state visual evoked potentials.

[26] Bin G., Gao X., Wang Y., Hong B., Gao S. (2009). VEP-based brain-computer interfaces: time, frequency, and code modulations. *IEEE Computational Intelligence Magazine*.

[27] Kapeller C., Hintermüller C., Abu-Alqumsan M., Prückl R., Peer A., Guger C. A BCI using VEP for continuous control of a mobile robot.

[28] Wang H., Li T., Huang Z. Remote control of an electrical car with SSVEP-based BCI.

[29] Wang Y., Wang Y.-T., Jung T.-P. (2010). Visual stimulus design for high-rate SSVEP BCI. *Electronics Letters*.

[30] Gergondet P., Petit D., Kheddar A. Steering a robot with a brain-computer interface: impact of video feedback on BCI performance.

[31] Diez P. F., Mut V. A., Laciar E., Avila Perona E. M. (2014). Mobile robot navigation with a self-paced brain-computer interface based on high-frequency SSVEP. *Robotica*.

[32] Choi B., Jo S. (2013). A low-cost EEG system-based hybrid brain-computer interface for humanoid robot navigation and recognition. *PLoS ONE*.

[33] Tidoni E., Gergondet P., Kheddar A., Aglioti S. M. (2014). Audio-visual feedback improves the BCI performance in the navigational control of a humanoid robot. *Frontiers in Neurorobotics*.

[34] Zhao J., Li W., Li M. (2015). Comparative study of SSVEP- and P300-based models for the telepresence control of humanoid robots. *PLoS ONE*.

[35] Rutkowski T. M., Shimizu K., Kodama T., Jurica P., Cichocki A. (2015). Brain-robot interfaces using spatial tactile BCI paradigms. *Symbiotic Interaction*.

[36] Brunner C., Allison B. Z., Krusienski D. J. (2010). Improved signal processing approaches in an offline simulation of a hybrid brain-computer interface. *Journal of Neuroscience Methods*.

[37] Gembler F., Stawicki P., Volosyak I. (2015). Autonomous parameter adjustment for SSVEP-based BCIs with a novel BCI wizard. *Frontiers in Neuroscience*.

[38] Sellers E. W., Vaughan T. M., Wolpaw J. R. (2010). A brain-computer interface for long-term independent home use. *Amyotrophic Lateral Sclerosis*.

[39] Holz E. M., Botrel L., Kübler A. Bridging gaps: long-term independent BCI home-use by a locked-in end-user.

[40] Friman O., Volosyak I., Gräser A. (2007). Multiple channel detection of steady-state visual evoked potentials for brain-computer interfaces. *IEEE Transactions on Biomedical Engineering*.

[41] Volosyak I., Malechka T., Valbuena D., Gräser A. A novel calibration method for SSVEP based brain-computer interfaces.

[42] Volosyak I., Cecotti H., Gräser A. Optimal visual stimuli on LCD screens for SSVEP based brain-computer interfaces.

[43] Wolpaw J. R., Birbaumer N., McFarland D. J., Pfurtscheller G., Vaughan T. M. (2002). Brain-computer interfaces for communication and control. *Clinical Neurophysiology*.

